# Systemic immunometabolism and responses to vaccines: insights from T and B cell perspectives

**DOI:** 10.1093/intimm/dxad021

**Published:** 2023-06-18

**Authors:** Sam Nettelfield, Di Yu, Pablo F Cañete

**Affiliations:** Frazer Institute, Faculty of Medicine, University of Queensland, Brisbane, Queensland 4072, Australia; Frazer Institute, Faculty of Medicine, University of Queensland, Brisbane, Queensland 4072, Australia; Ian Frazer Centre for Children’s Immunotherapy Research, Child Health Research Centre, Faculty of Medicine, University of Queensland, Brisbane, Queensland 4072, Australia; Frazer Institute, Faculty of Medicine, University of Queensland, Brisbane, Queensland 4072, Australia; Ian Frazer Centre for Children’s Immunotherapy Research, Child Health Research Centre, Faculty of Medicine, University of Queensland, Brisbane, Queensland 4072, Australia

**Keywords:** elderly, malnutrition, metabolic hormones, obesity, T cells

## Abstract

Vaccination stands as the cornerstone in the battle against infectious diseases, and its efficacy hinges on several host-related factors like genetics, age, and metabolic status. Vulnerable populations, such as malnourished individuals, the obese, and the elderly, commonly exhibit diminished vaccine responses and efficacy. While the specific factors contributing to this impairment may vary, these individuals typically display a degree of metabolic dysregulation, thereby underscoring its potential significance as a fundamental determinant of suboptimal vaccine responses. The emerging field of immunometabolism aims to unravel the intricate interplay between immune regulation and metabolic pathways, and recent research has revealed diverse metabolic signatures linked to various vaccine responses and outcomes. In this review, we summarize the major metabolic pathways utilized by B and T cells during vaccine responses, their complex and varied metabolic requirements, and the impact of micronutrients and metabolic hormones on vaccine outcomes. Furthermore, we examine how systemic metabolism influences vaccine responses and the evidence suggesting that metabolic dysregulation in vulnerable populations can lead to impaired vaccine responses. Lastly, we reflect on the challenge of proving causality with respect to the contribution of metabolic dysregulation to poor vaccine outcomes, and highlight the need for a systems biology approach that combines multimodal profiling and mathematical modelling to reveal the underlying mechanisms of such complex interactions.

## Introduction

Vaccination represents one of the most important public health tools in the fight against infectious diseases, and the success of vaccines is largely reliant on the ability of the adaptive immune system to generate long-lasting cellular and humoral immunity. Although several host-extrinsic factors such as vaccine design, pathogen biology and environmental conditions can impact the extent of immune protection afforded by vaccination, host-related variables also play a critical role in determining vaccine efficacy. Notably, genetic variation, age-related changes and metabolic states are all key determinants of vaccine efficacy.

The emerging field of immunometabolism seeks to elucidate the interplay between immune regulation and metabolic landscapes, and how these interactions can be harnessed to improve health outcomes. A recent multiscale, multifactorial response network analysis demonstrated that metabolic changes as early as 1 day after vaccination were correlated with vaccine immunity (1). However, whilst recent studies have revealed a diverse range of metabolic signatures associated with SARS-CoV-2 vaccination, whether these states can serve as reliable predictors or inform vaccine design remains uncertain (2–4).

Systemic metabolism refers to the collection of all biochemical reactions that occur within an organism, and it comprises a vast array of pathways that constitute the backbone of life. These pathways comprise numerous biochemical reactions, transport processes, metabolites, and cofactors that facilitate the synthesis or breakdown of organic compounds while providing the energy required to sustain life. There has been an exponential growth in the field that strives to understand cellular metabolism in the context of immunity beyond merely generating energy and building cellular components. Indeed, in addition to their bioenergetic and biosynthetic functions, metabolites also serve as essential communication signals that can respond to environmental cues. In the context of immunology, these signals are typically sensed by central pathways like the mammalian target of rapamycin (mTOR), which integrates environmental information such as nutrients to coordinate metabolic adaptations, orchestrate transcriptional networks, influence cellular proliferation, and drive effector functions. These adaptations are particularly critical for the immune system, with up to 30% of the organism’s basal metabolic rate estimated to be devoted to adaptive immune system activation alone (5).

It is widely acknowledged that people with metabolic perturbations, such as malnourished or obese individuals or the elderly, often exhibit suboptimal humoral responses to most vaccination regimes (6, 7). Such individuals may experience abnormal micronutrient levels, metabolic imbalances or altered metabolic hormone levels, all of which can affect vaccine outcomes. In this review, we will examine how metabolic signatures characteristic of various T and B cell subsets drive adaptive immune responses. We will also outline the immunomodulatory properties of key micronutrients and metabolic hormones. Finally, we will discuss the impact of malnutrition, obesity, and ageing on adaptive immunity and vaccine efficacy and reflect on potential avenues for future research aimed at enhancing vaccine responses in these populations. The regulation of innate immune responses following vaccination is also increasingly recognized to be influenced by cellular and systemic metabolism, as supported by emerging evidence demonstrating that a subset of cells with the capacity for memory-like differentiation undergoes extensive metabolic reprogramming (8, 9).

## Cellular metabolism of T and B cells

**Fig. 1.**
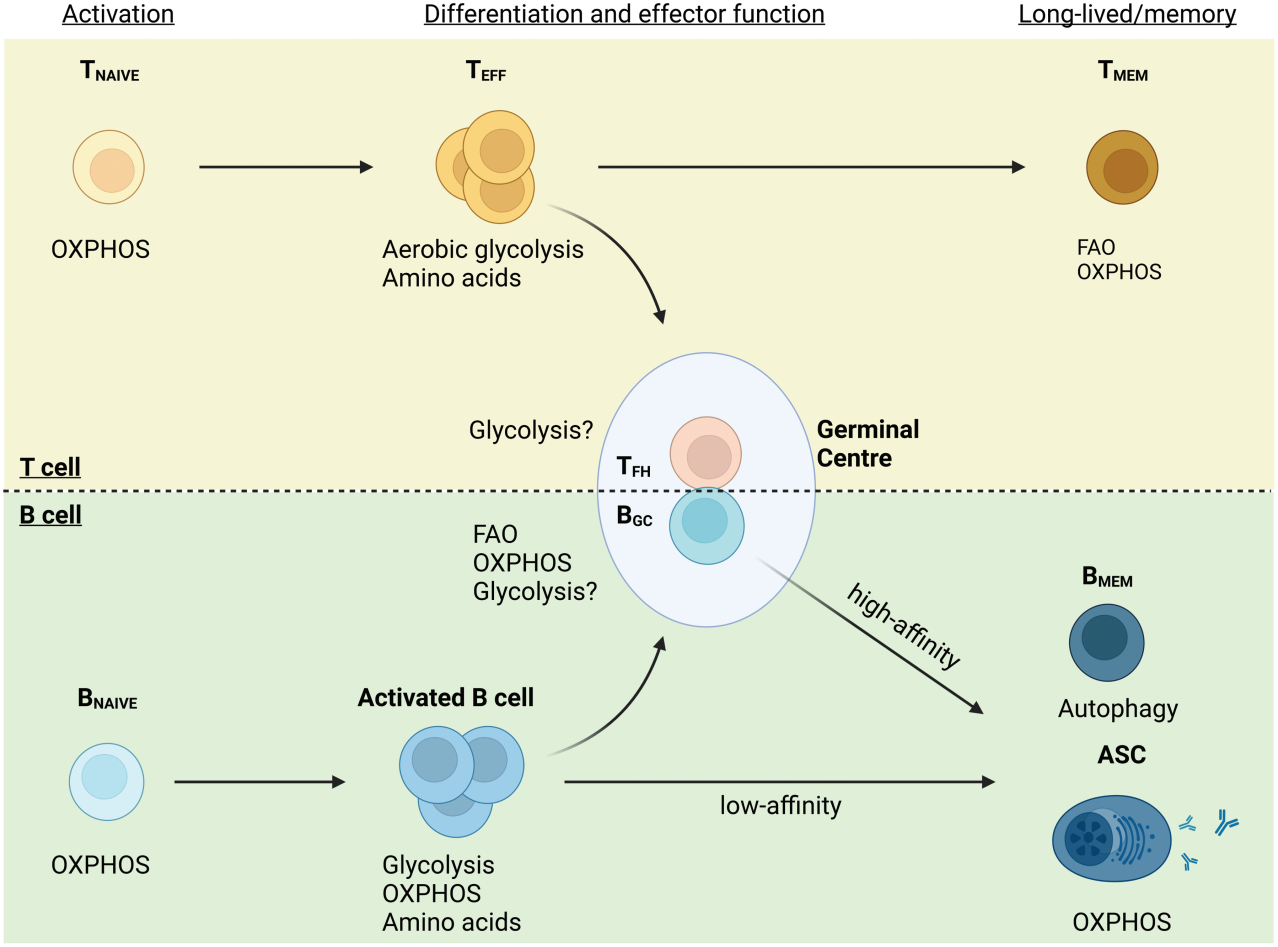
T and B cells undergo metabolic reprograming during an immune response. Prior to antigen recognition, T and B cells exist in a metabolically quiescent state utilizing oxidative phosphorylation (OXPHOS). Upon immune challenge, T cells and B cells undergo rapid expansion and perform unique effector functions, each adopting unique metabolic programs. Key to the development of high-affinity antibodies, a small fraction of effector T (T_EFF_) cells and activated B cells go on to form germinal centres and develop into follicular helper T (T_FH_) cells and germinal centre B (B_GC_) cells, respectively. Following antigen clearance, T cells can develop into memory T (T_MEM_) cells whereas B cells can develop into either memory B (B_MEM_) cells that utilize autophagy to ensure longevity or into long-lived antibody-secreting cells (ASCs).

Lymphocytes are the cornerstone of adaptive immune responses. In essence, antigen recognition by naive B or T cells triggers activation and subsequent stepwise differentiation into effector or memory cells. Such differentiation routes are complex and pose unique metabolic requirements for lymphocytes, even more so when considering the breadth of cellular states that a single T or B cell may experience during its lifetime. For instance, a single mature lymphocyte that has never encountered antigen typically resides in a naive quiescent state. Upon antigen encounter, and under the right conditions, this same cell may (i) transition to an activated state with effector functions, (ii) undergo intense proliferative bursts—entering and exiting cell cycle, and (iii) be subjected to one of multiple specialized differentiation routes depending on the context of the immune response. Finally, upon antigen clearance most clones will undergo programmed cell death, and those who survive then return to a quiescence-like memory state. Therefore, a single lymphocyte’s lifetime is dynamic and complex, and it entails entering multiple cellular states with varying energy requirements and metabolic activity (Fig. 1).

### Lymphocyte activation

T cells are essential players in coordinating multiple aspects of adaptive immunity, including responses to pathogens, allergens and tumours. During these processes, T cells regulate and adapt their metabolism in response to changing antigen-driven and/or microenvironmental signals. Naive T cells maintain cellular homeostasis with relatively low energy demands, relying heavily on oxidative phosphorylation (OXPHOS) fuelled by the tricarboxylic acid (TCA) cycle. However, upon T cell receptor (TCR) engagement and co-stimulatory signals, T cells shift to the ATP-inefficient Warburg metabolism with reduced OXPHOS and increased aerobic glycolysis. Following antigen clearance, T cells can transition into long-lived memory cells with similar metabolic rates to naive cells, but with enlarged mitochondria and increased lipid usage together with fatty acid oxidation (FAO)—thought to facilitate rapid activation upon antigen re-counter (10, 11).

B cell metabolism is remarkably different from that of T cells because of the nature of the antibody response. The primary role of a B cell is to develop into antibody-secreting cells (ASCs), which are essentially protein factories that continuously produce hundreds to thousands of antibody molecules per second. Thus, this requires not only increased proliferation, but also extensive protein turnover to fuel the anabolic process of antibody production and secretion. The energy demands of activated B cells are largely met by increasing the ATP-efficient glycolysis–OXPHOS pathway (12). Additionally, B cells utilize alternative carbon sources (in addition to glucose) such as amino acids and fatty acids to fuel OXPHOS. Following activation, B cells develop into either ASCs or memory B (B_MEM_) cells. In ASCs, glucose breakdown via the hexosamine pathway has been shown to be essential for glycosylation of antibodies, whereas pyruvate used in OXPHOS is largely sourced from FAO (13).

Even though the goal of vaccination is to generate long-lived ASCs and memory B cells, the metabolic intricacies underlying the latter remain poorly understood. Emerging evidence highlights that autophagy in B_MEM_ cells determines their longevity and thus contributes to vaccine-mediated protection (14). Other B cells that adopt the follicular differentiation route have been shown to exhibit unconventional metabolic requirements. For instance, germinal centre B (B_GC_) cells, which undergo multiple rounds of somatic hypermutation and T cell-mediated affinity selection, appear to be largely dependent on a variety of metabolic processes.

### B and T cell collaboration

The primary objective of the germinal centre (GC) reaction is to generate long-lived, high-affinity ASCs or B_MEM_ cells (15, 16). The success of vaccination strategies is heavily reliant on the production of durable and potent antibodies, necessitating a comprehensive understanding of the metabolism of follicular helper T (T_FH_) cells and B_GC_ cells (17). Recent evidence has shown that T_FH_ cells exhibit elevated glycolytic activity, which is crucial for their differentiation (18, 19). However, the T_FH_ master transcription factor, Bcl6, is known to directly suppress certain components of the glycolytic pathway (20). For instance, studies conducted in DUSP6^−/−^ mice, which lack a protein essential in TCR-mediated upregulation of glycolysis, suggest that a reduction in glycolysis leads to increased T_FH_ cell counts and greater expression of the canonical T_FH_ cytokine IL-21 (21). Additionally, T_FH_ cells are susceptible to ferroptosis, a form of cell death triggered by iron-dependent accumulation of lipid peroxidation, which is tightly linked with cellular oxidative stress (22, 23). Numerous metabolic pathways unique to T_FH_ cell metabolism are emerging, likely reflective of the unique competitive environment within the GC.

GCs present a challenging area of research when it comes to understanding the metabolic dynamics of its key cellular players. Within GCs, B cells undergo a series of iterative cycles of proliferation within the dark zone, followed by antigen presentation and affinity-based selection in the hypoxic light zone upon interactions with cognate T_FH_ cells. Research in this area has been constrained by challenges associated with detecting these events *in vivo*. The highly dynamic nature of GCs, the staggering brief half-life of B_GC_ cells and the difficulty in discerning positive versus negative selection, as well as differentiation versus cyclic re-entry, present significant obstacles. Nevertheless, recent *ex vivo* analysis of *bona fide* B_GC_ cells suggests a predominant reliance on OXPHOS largely driven by FAO (24). This contradicts other reports indicating a dependence on the mTOR signalling pathway (25, 26), which may implicate glucose metabolism, although evidence remains limited. Nonetheless, the intricate and incompletely understood metabolic landscape within GCs hinders efforts to establish links between metabolic phenotypes and suboptimal vaccine responses, as well as the development of vaccine design strategies.

### Micronutrients support T and B cells responses during vaccination

**Fig. 2.**
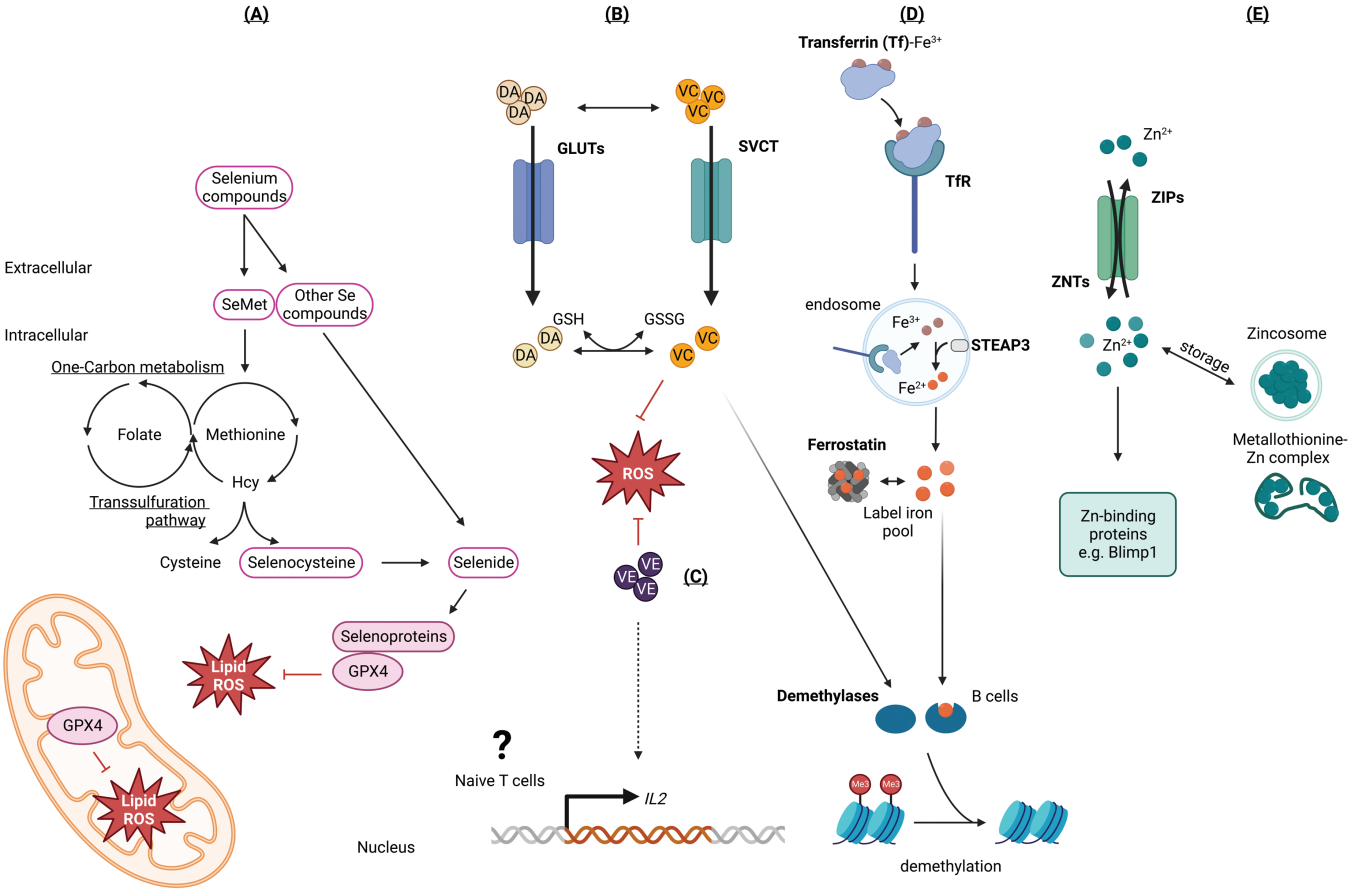
Micronutrients support immune function through various metabolic pathways. Vitamins and trace elements are important determinants of vaccine outcomes by shaping functional states of T and B cells. (A) Selenium (Se) metabolism culminates in the generation of selenoproteins that mediate essential cellular functions. (B) Vitamin C (VC) and (C) vitamin E are potent antioxidants and affect cellular metabolism through various effect. (D) Iron (Fe^3+^) enters the cell by receptor-mediated endocytosis where it is reduced to soluble iron (Fe^2+^) via the metalloreductase STEAP3 and forms the labile iron pool to exert its effects. (E) Zinc (Zn^2+^) enters via transporters such as ZIPs and ZNTs and exerts its function through numerous zinc-binding proteins including Blimp-1, which is an antibody-secreting cell master transcription factor. Hcy, homocysteine; α-KA, α-keto acid; α-KG, α-ketoglutarate; AA, amino acid; DA, dehydroascorbate; GLUT, glucose transporter; SVTC, sodium-ascorbate co-transporters; GSH, glutathione; GSSH, glutathione disulfide; ROS, reactive oxygen species.

In addition to glucose, T cells also utilize amino acids, such as glutamine, and fatty acids derived from the extracellular matrix to meet their energy demands during activation (27, 28). A variety of micronutrients, such as vitamins E and C have also been shown to control important aspects of T and B cell function (29) (Fig. 2). Vitamin E has been shown to drive expression of IL-2 specifically in naive T cells (30), while deficiency of vitamin E in mice and other mammals results in impaired immune responses that can be rescued upon supplementation. Likewise, several reports have observed that vitamin E supplementation in vaccinated individuals increases lymphocyte numbers and reduces oxidative stress (31). In the context of B cells, vitamin C (ascorbic acid) has been shown to promote plasma-cell differentiation directly within B_GC_ cells, via demethylation of Blimp-1 enhancer regions (32).

Similarly, trace elements such as zinc, iron and selenium have emerged as key regulators of T and B cell functions. Notably, recent investigations have unveiled a compelling association between iron deficiency and compromised antibody responses following vaccination in both human and mice. Mechanistically, cellular iron (Fe^2+^) directly facilitates histone demethylase activity, which is essential for B cell proliferation (33). Similarly, a diet-induced low serum iron concentration has been demonstrated to reduce T and B cell numbers within the GC and decrease antigen-specific IgG antibodies during immunization and infection (34). Selenium, another trace element with cellular functions primarily linked to selenoproteins (35), appears to be fundamental for facilitating antibody responses and vaccine efficacy. Specifically, selenium has been shown to be indispensable for T_FH_ cells, where it acts as a cofactor for glutathione peroxidase 4 (GPX4), effectively mitigating cell death induced by reactive oxygen species (ROS) generated during intracellular lipid metabolism (22). As such, selenium administered as a dietary supplement (22, 36) or vaccine adjuvant (37) has been observed to increase antibody responses to vaccination and improve vaccine efficacy, at least partially through the mitigation of ferroptosis in T_FH_ cells.

Intriguingly, selenium supplementation has been shown to regulate the function of T_H_17-like cell types (38). Given that T_H_17-type T_FH_ cells (also called T_FH_17) outcompete T_FH_1 or T_FH_2 cells in maintenance and memory function in vaccination (39), whether and how selenium specifically influences T_FH_17 homeostasis and function will be an important question to address. Overall, these studies highlight the critical role that micronutrients, such as vitamin E and C, zinc, iron, and selenium, play in regulating T and B cell responses and vaccine outcomes, and suggest that supplementing with these micronutrients may improve vaccine efficacy.

### Regulation of T and B cells by metabolic hormones

**Fig. 3.**
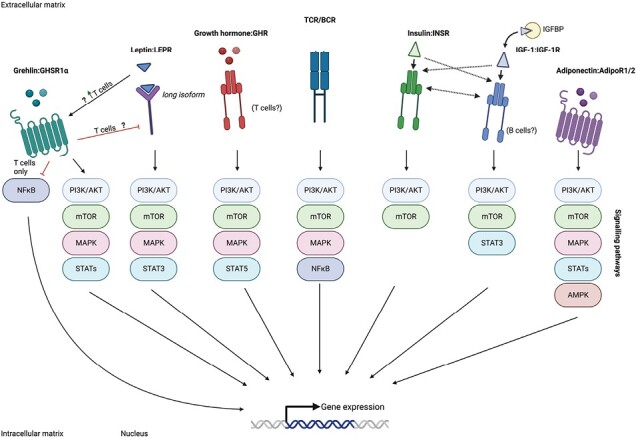
Metabolic hormones regulate T and B cells through major signalling pathways. Upon TCR/BCR activation, metabolic hormones are often increased in response to elevated energy demand and metabolic requirements. Typically, metabolic hormones promote T and B cell activity by increasing their proliferation, function, or survival. Insulin and insulin-like growth factor 1 (IGF-1) are evolutionarily and structurally related, and thus both receptors recognize and bind to their respective ligand, albeit with reduced affinity. Moreover, a hybrid receptor composed of insulin and IGF-1 dimers signals through both insulin and IGF-1 signalling cascades. GHSR1α; growth hormone secretagogue receptor 1α; LEPR, leptin receptor; GHR, growth hormone receptor; TCR, T cell receptor; BCR, B cell receptor; INSR, insulin receptor; IGFBP, IGF-binding protein.

Metabolic hormones, such as leptin, insulin, ghrelin, and growth hormone, which typically serve as chemical messengers of the endocrine system, have been found to play fundamental roles in the regulation of immune cell function (9, 40). We now know that immune cells can express a plethora of membrane receptors that bind to metabolic hormones, and signalling through these receptors modulates their function, proliferation and cell fate (Fig. 3). Hence, investigating whether metabolites can serve as predictors of vaccine efficacy is an intriguing area of research. As the precise regulatory role of metabolic hormones is becoming better understood, further work is needed to fully elucidate how metabolic hormones impact T and B cells and regulate vaccine responses.

Metabolic hormones have recently been shown to have a direct role on T cell biology, providing a key link between organismal energy status and cellular metabolism. T cell activation leads to an increase in energy demands that requires metabolic reprogramming. This reprogramming is often associated with increased expression of cell surface hormone receptors such as the classical receptor for leptin (leptin receptor, LEPR), insulin (insulin receptor, INSR) and ghrelin (ghrelin hormone secretagogue receptor 1α, GHSR1α). Upregulation of these receptors also promotes T cell activation either *in vitro* and *in vivo* (41–44), with leptin–LEPR and insulin–INSR leading to increased glucose metabolism in CD4^+^ T cells (44, 45). Indeed, T cell specific deletion of INSR (*Insr*^*fl/fl*^:*Lck*.Cre) leads to a reduction in antigen-specific T cell numbers *in vitro* and during protein immunization and infection (45). Moreover, INSR^−/−^ T cells also display defective cytokine responses *in vivo*, and exhibit a stark decrease in IFN^+^, TNF^+^ and IL-17^+^ effector T cells (45). Similar results have also been shown in the context of leptin by use of *in vitro* assays in humans (46) and, interestingly, addition of ghrelin abrogated all these effects (46), highlighting the antagonistic relationship between leptin and ghrelin in T cells. Moreover, inhibition of autocrine ghrelin signalling in T cells leads to increased NFκB signalling and pro-inflammatory cytokine secretion, further supporting a suppressive role of ghrelin in T cell function (47).

In addition to warping T cell function and activation, metabolic hormones have also been shown to influence T cell differentiation. For example, the differentiation of T_H_17 cells has been shown to be dependent on multiple hormone receptor interactions including leptin–LEPR (48), insulin-like growth factor 1 (IGF-1)–IGF-1R (49) and insulin–INSR (45) while also being inhibited by ghrelin–GHSR1α signalling (41).

In comparison to T cells, regulation of B cells by metabolic hormones is less understood with only limited B cell intrinsic studies having been made. Recent studies have revealed a role of adiponectin in promoting inflammatory responses through B cells specifically in the context of arthritis. One recent study has shown that adiponectin activates PI3K, an energy-driven signalling pathway, which promotes plasma-cell differentiation within the inflamed synovial joint tissue of a collagen-induced arthritis mouse model (50). Another study revealed a novel mechanism through which B cells inhibit T cell migration across endothelial cells. This process involves the tonic secretion of a soluble peptide controlled by adiponectin receptor expression on B cells. Notably, in inflammatory conditions such as rheumatoid arthritis, expression of adiponectin receptor on B cells is reduced, thereby abrogating tonic inhibition of T cell migration and increasing disease pathogenesis (51). Additional immunoinhibitory B cell-mediated functions have been described by Frasca *et al.* (52), whereby leptin inhibits the development of antigen-specific IgG plasma-cells and invigorates expression of inflammatory markers in B cells.

Despite the growing recognition of metabolic hormones’ influence on T and B cell biology, unravelling the intricate molecular mechanisms governing metabolic regulation of these cells during vaccine responses remains a challenge. A recent pivotal study sheds light on the role of leptin signalling, in both human and murine T_FH_ cells, on the generation of high-affinity antibodies (53). Mechanistically, the authors demonstrate that leptin promotes IL-21 production (53), which in turn supports B_GC_ cell selection and prioritizes ASC differentiation (54). Therefore, leptin emerges as a potential regulator of T_FH_ cell functionality. Another metabolic hormone, IGF-1, directly impacts vaccine efficacy, as shown in a mouse model of macrophage-specific IGF-1 deficiency (*Igf1*^*fl/fl*^*:Lyz2*.Cre), which exhibited a significant reduction of antigen-specific IgG titres following influenza vaccination (55). Collectively, these studies provide critical insights into the intricate field of immunometabolism, illuminating the crucial role of hormonal regulation in antibody responses and vaccine outcomes.

## Systemic metabolism and vaccine responses

### Malnutrition

Given the complex and dynamic metabolic requirements needed to coordinate immune responses, several lines of evidence suggest that metabolic dysregulation at the organismal level can have detrimental effects on vaccination outcomes. For instance, malnutrition is a complex problem with diverse consequences, encompassing several nutritional deficiencies, including intrauterine growth restriction, stunting, wasting, suboptimal breastfeeding and a lack of micronutrients such as vitamin A and zinc (56). Since each of these manifestations increases the risk and/or severity of infections, malnutrition has been identified as the most prevalent cause of immunodeficiency globally (57). Metabolic dysregulation can arise in the context of malnutrition, disrupting the balance of hormones and nutrients that regulate metabolism. In response to nutrient deprivation, the body conserves energy through a slowdown in metabolism, leading to imbalances in hormones like insulin and leptin, which can ultimately lead to immune dysfunction. Consistent with this, we have previously observed that leptinaemia, which is associated with malnutrition or undernutrition, appears to be a risk factor for lower antibody responses in influenza-vaccinated individuals (53).

The relationship between malnutrition and infection is bidirectional, with infections leading to malnutrition through reduced intake and absorption of nutrients, as well as the diversion of nutrients away from growth (58). Not surprisingly, malnutrition often leads to a preponderance of severe infections in affected individuals. Interestingly, although T cell numbers appear to be mostly unaffected in malnourished individuals, thymic involution is a hallmark of malnutrition (59). Indeed, even mildly malnourished children exhibit a compromised thymus and, given its role in carrying out T cell education, it is possible that these individuals may contain incomplete or suboptimal T cell repertoires. This would explain the immunodeficient phenotypes observed across all incidences of malnourishment. Nevertheless, the field has long recognized an apparent paradox in malnourished children who, despite being severely immunocompromised and often dying from infections, still appear to mount appropriate immune responses to vaccines.

There is a wealth of data generated by various studies that have evaluated the responses of malnourished children to different types of vaccines, such as diphtheria and tetanus, as well as hepatitis B, which indicate that they generally respond adequately to protein vaccines albeit with subtle decreases in antibody affinity or titre (60). However, given that these studies have mostly focussed on short-term antibody responses, it is possible that they may underestimate the impact of malnutrition on the quality or duration of the immune response. Alternatively, problems with mucosal barriers and gut dysbiosis associated with malnutrition may lead to infectious illness and death, while the body’s response to vaccines remains relatively strong.

Attributing immune dysfunction phenotypes solely to metabolic dysregulation in malnutrition presents a challenge, as malnutrition encompasses a complex interplay of several factors such as infection, inflammation, mucosal barrier dysfunction, immune dysregulation, inappropriate growth, and nutritional status. Untangling the specific contribution of metabolic dysregulation from any of these factors to the development of malnutrition-associated immune dysfunction is thus challenging. However, gaining a deeper understanding of how nutrient-sensing pathways impact cellular function within the context of malnutrition can offer valuable insights into its development and progression. Such insights can also guide potential interventions aimed at combating infections and addressing immune deficiencies in individuals affected by malnutrition.

### Obesity

**Fig. 4.**
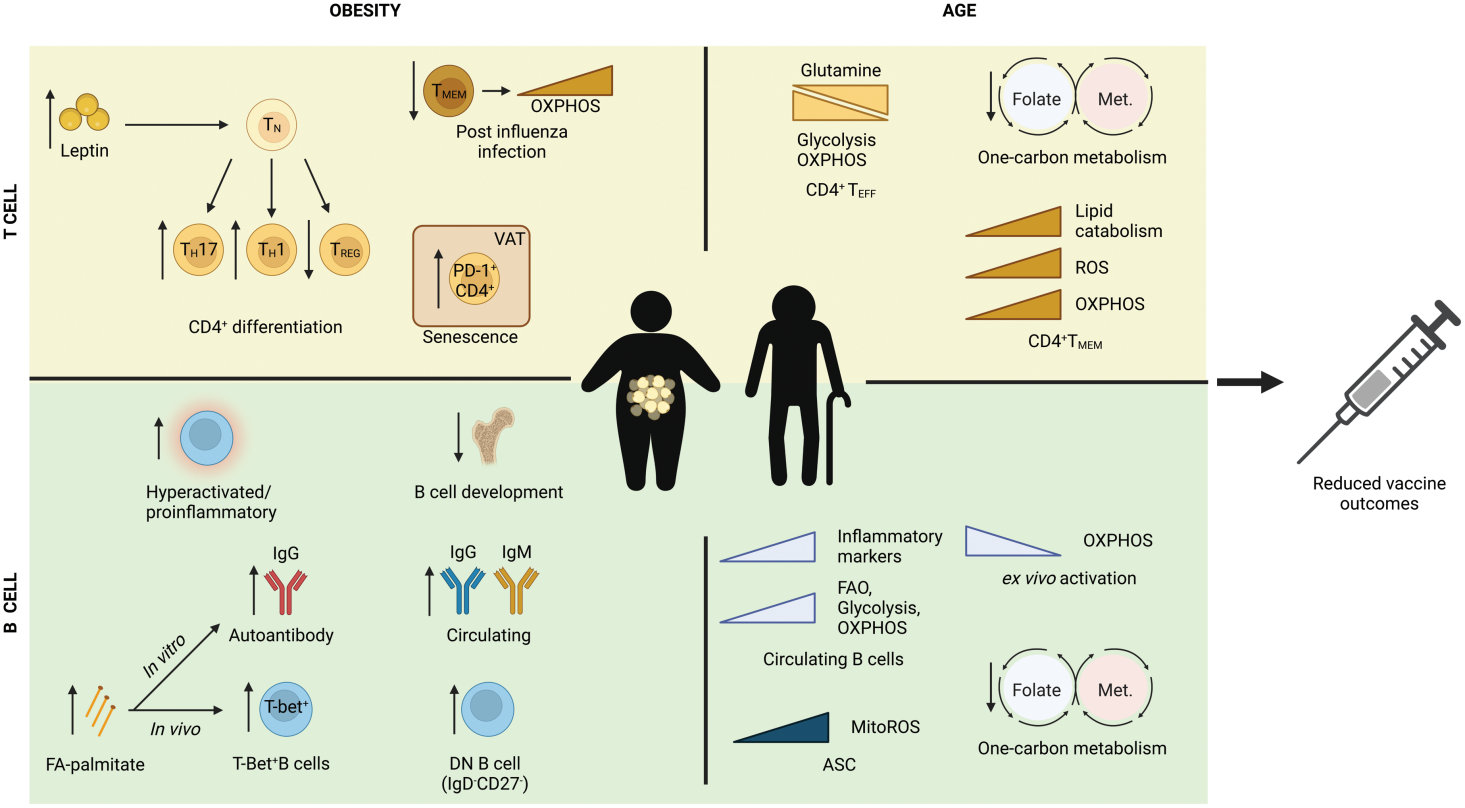
Obesity (left) and age (right) are associated with metabolic changes within T (top) and B (bottom) cells that may contribute to poor vaccine outcomes. Obesity is a metabolically dysregulated environment associated with increased inflammation, hormone resistance and increased levels of metabolites and adipokines. In this environment, T cells and B cells preferentially develop into pro-inflammatory effector cells that contribute towards systemic inflammation and autoantibody production. In an aged immune system, T and B cell responses wane, likely attributed to immunoscenescence. Although a conclusive causal link between waning immune responses and age has yet to be made, numerous cellular metabolic processes have been identified within T and B cells that may contribute towards this phenotype. Together, obesity and age present a metabolically distorted environment that dysregulates T and B cells that renders them ineffective in providing effective vaccine outcomes. T_N_, naive T cell; T_H_, helper T cell; T_REG_, regulatory T cell; T_EFF_, effector T cell; DN B cell, double negative B cell; ASC, antibody-secreting cell; Met., methionine.

More recent studies have further elucidated the impact of metabolic dysregulation on vaccine responses, particularly in the context of obesity and type 2 diabetes (Fig. 4). Excess adipose tissue in obese individuals can lead to chronic low-grade inflammation, which can disrupt normal metabolic processes and contribute to insulin resistance, a hallmark of metabolic dysfunction. Adipose tissue produces several hormones, including adiponectin, leptin and resistin, which regulate energy homeostasis, appetite, glucose metabolism and inflammation. Elevated levels of such hormones induce a state of metabolic dysfunction, triggering the secretion of excessive pro-inflammatory cytokines that recruit immune cells. In conjunction with these immune infiltrates, the release of inflammatory factors ensues, thus facilitating chronic low-grade inflammation that hampers effective immune responses (61, 62).

Obese individuals manifest some perturbations within the T cell compartment. Elevated pro-inflammatory effector T cells with a concomitant reduction in regulatory T cells has been documented in several reports (63, 64). Such changes can be attributed to, at least in part, an increase in serum leptin levels. Moreover, following influenza infection, obese individuals exhibit reduced numbers of memory T cells accompanied by abnormal metabolic signatures, mirrored by an increase in visceral adipose tissue resident T cell senescence (65, 66). Similarly, obesity is associated with a hyperactive B cell phenotype (67) and an increased predisposition to autoantibody production. Indeed, obese individuals exhibit elevated double negative B cells (68), which are thought to contribute and/or mediate disease pathogenesis in various autoimmune diseases and chronic inflammatory conditions (69).

There seems to be a consensus amongst researchers to indicate that obese individuals are associated with poor immune responses. There is a wealth of evidence suggesting that obesity leads to perturbed immune responses with unfavourable outcomes, and thus it is not surprising that decreased seropositive convertion rates are common in obese individuals following vaccination. Specifically, poor antibody responses have been reported in obese individuals upon tetanus (70), influenza (66, 71) or inactivated SARS-CoV-2 (72) vaccinations. In contrast, other vaccination regimes, such as live-attenuated hepatitis A, have been shown to be equally effective in both obese and lean individuals, suggesting that other factors may be at play (73). Furthermore, several studies have observed lower antibody titres upon influenza vaccination in obese individuals, and this defect is typically mirrored by decreased protection from influenza-related complications compared with non-obese individuals (74, 75).

The relationship between obesity and vaccine responses is a topic of ongoing research and controversy. Although some studies have found conflicting results on whether obesity impairs antibody responses to vaccines such as influenza, there is a general consensus among researchers that obese individuals are at a higher risk for developing severe disease. This phenomenon has been observed across several large longitudinal studies, and its significance entails poor vaccine protection despite abundant antibody titres upon vaccination. Thus, it is tempting to speculate that antibody titres may not be the best predictors to gauge vaccine protection. This increased risk is thought to be related to the chronic inflammation and altered immune function associated with obesity, which can impair the body’s ability to fight off infections (66, 76). However, whether this risk is a result of metabolic dysregulation is still unclear, as obesity is often accompanied by other comorbidities like cardiovascular disease, which can further increase the risk of severe disease and mortality, particularly in the context of respiratory infections.

Overall, although the relationship between obesity and vaccine responses is complex and not fully understood, it is clear that obesity is associated with an increased risk of severe disease from infectious diseases. As such, it is imperative to elucidate the underlying mechanisms behind these unfavourable outcomes. Unravelling the intricate metabolic pathways and molecular drivers involved will not only enhance our understanding but also facilitate the refinement and development of targeted vaccination strategies tailored specifically to address the unique immunological challenges presented by obesity.

### Ageing

Aged individuals are a clinically vulnerable cohort associated with metabolic changes that are accompanied by poor vaccine outcomes (Fig. 4), including Japanese encephalitis (77) and influenza (6). An ageing/aged immune system has been shown to lead to a variety of immunological changes (Table 1) and is thought to elicit poor vaccine-induced immunity becuase of immunosenescence (98). Indeed, long-term efficacy studies of SARS-CoV-2 BNT162b2 have reported a lack of detectable neutralizing antibodies in ~20% of participants aged 70–89 years, mirrored by prevalent antigen escape against multiple variants after 20 weeks (99). Similarly, aged mice and non-human primates exhibit defective GC responses (100, 101), providing a mechanism for age-associated reduced vaccine-mediated immunity. Understanding the relationship between age-related metabolic changes and immune function is challenging because of numerous confounding variables. Although it is clear that aged individuals mount poor immune responses, the contribution of metabolic changes to this defect remains elusive.

**Table 1.** Age-associated effects on T and B cells.

Role	Cell subset	Model	Main effect of ageing	References
T cells				
Steady-state and activation	Total T cells	Human (28–85 years)	Naive T cell TCR repertoire contracted and is more evident in CD8^+^ T cells	(78)
		Human (20–90 years)	Significantly reduced number of CD4^+^ and CD8^+^ T cells	(79)
		Mouse young (1 month) and old (18 months)	CD4^+^ and CD8^+^ T cells have altered transcriptional landscape	(80)
	CD4^+^ T cells	Mouse young (2–3 months) and old (18–20 months)	Increased apoptosis *in vitro*, decrease frequency to transition to central memory cells	(81)
	CD8^+^ T cells	Mouse young (4–6 months) and old (20–22 months)	Naive CD8^+^ T cells fail to maintain quiescence and transition to virtual memory cells instead	(82)
Differentiation and effector function	CD4^+^ T cells T_FH_ cells	Mouse young (2–4 months) and old (>15 months)	Reduced T-dependent B cell help leading to reduced humoral response	(83)
		Mouse young (8–12 weeks) and old (>85 weeks)	Increase in pre-T_FH_ cells (CD4^+^PD1^int^CXCR5^int^) with no change in GC-T_FH_ cells (CD4^+^PD1^hi^CXCR5^hi^)	(84)
		Mouse young (< 6 months), middle age (12–15 months) and old (>18 months)	Increase in IL-10 T_FH_ cells and administering IL-10-blocking antibody enhancing humoral response during immunization	(85)
	CD8^+^ T cells	Mouse young (3–5 months) and old (17-22 months)	Reduced IL-2 expression upon *in vitro* activation	(86)
		Mouse young (7 months) and old (19 months), and young (6–8 weeks) and old (21–22 weeks)	Decrease in proliferation of virus-specific CD8^+^ T cells	(86, 87)
Memory	Total T_MEM_	Mouse young (2 months) and old (18 months)	CD4^+^ T_MEM_ cells express higher cell death-associated genes while CD8^+^ T_MEM_ cells exhibit reduced expression	(80)
	CD4^+^ T_MEM_ cell	Mouse young (2–4 months) and old (14–16 months)	Reduced proliferation, cytokine production and helper function from T_MEM_ cells generated from aged naive cells *in vitro* and reduced humoral response *in vivo*	(88)
	CD8^+^ T_MEM_	Mouse young (2–4 months) and old (18–22 months)	Reduced proliferation potential and ability to mount recall response	(89)
B cells				
Steady-state and activation	Total B cells	Human children, young, adult (65–70 years), elderly (<70 years)	Reduced number of total B cells	(90)
		Mouse young (6 weeks) and old (> 32 weeks)	Reduced number of precursor B cells and IL-7R expression	(91)
		Human young (20–34 years) and old (>64 years)	No change in activation markers *in vitro*	(92)
		Human young (18–45 years) and old (61–68 years)	Repertoire diversity of B cells reduced in blood and lymph nodes but increased in spleen	(93)
	Plasma-cell	Human cord and peripheral blood (newborn to > 80 years)	Reduced number of plasma-cells	(94)
Differentiation and effector function	Activated B cell	Mouse young (2–4 months) and old (24–27 months)	*In vitro* activated senescent B cells have reduced class-switch recombination	(95)
		Human children, young, adult (65–70 years), elderly (<70 years)	Reduced IgM and IgA production from T-independent stimulation	(90)
	Germinal centre B cells	Immunized mouse model receiving adoptive transfer of cells from young (10 weeks) or aged (93 weeks) mice	Preference for extrafollicular development over GC entry early post-immunization	(92)
		Human young (20–34 years) and old (68–76 years), mouse young (10 weeks) and old (93 weeks)	Delayed early germinal centre kinetics *in vivo*; however, no decline in antibody secretion from both naive and memory B cells *in vitro*	(92)
Memory	Memory B cell	Human young (23–40 years) old (>75 years)	Reduced number of total B_MEM_ cells	(96)
		Human peripheral blood (18–86 years)	Reduced number and frequency of switched memory B cells (CD19^+^IgG^+^IgA^+^)	(97)

Recent studies have provided new insights into the underlying metabolic differences that may lead to dysregulated B cell responses and vaccination. A study by Kurupati *et al.* (102) compared metabolic signatures in B cells with vaccine responses following trivalent influenza vaccination and found that the elderly had reduced antibody titres against H1N1 and H3N2 strains and reduced expression of SIRT1. SIRT1 is an NAD-dependent deacetylase involved in various functions, including plasma-cell differentiation and antibody responses, by integrating metabolic cues with epigenetic changes (103). Using both young and aged subjects, SIRT1 levels were shown to be reduced in poor vaccine responders (102). This study also went on to show that various aged B cells subsets in circulation had an increased mitochondrial ROS and mass, and a significant decrease in OXPHOS upon *in vitro* activation (102) indicative of an altered metabolic profile. Indeed, it was later shown by another group that aged circulating B cells have higher levels of glucose uptake and glycolysis leading to an overall increase in glucose metabolism (104). Moreover, these B cells had an increase in inflammatory markers (TNF, IL-6 and p16) and autoantibody production upon *ex vivo* activation (104), suggesting a skewed immune response.

Recent advancements have shed light on the metabolic mechanisms underlying the age-related decline in T cell function. There is some evidence suggesting that the metabolic profiles of aged and young effector CD4^+^ T cells differ (105). In the context of aged mice, T cells exhibit an augmented reliance on glutamine metabolism, alongside impairments in glycolysis and OXPHOS compared to their younger counterparts (105). Moreover, the biosynthetic one carbon metabolism pathway shown to be fundamental in T cell activation (106) and many other critical pathways, including amino acid and nucleotide synthesis, has been reported to be reduced in both T and B cells in ageing studies (102, 107). Whereas human CD4^+^ T cells from elderly individuals have been shown to have increased OXPHOS upon *in vitro* activation, they exhibit no difference in recall responses compared with young individuals (108). Collectively, these studies provide evidence of age-related metabolic modifications in various T cell subsets. However, it remains unclear whether these alterations are causative factors of age-related T cell dysfunction or rather a consequence of the ageing process.

There is a wealth of data describing numerous metabolic changes during ageing with a concomitant decline in immune function, including impaired T cell responses. However, a recent elegant 10-year longitudinal study has provided compelling evidence demonstrating the intrinsic capacity of T cells for virtually unlimited population expansion, surpassing the life-span of their host organism (109). These long-lived T cells, referred to as induced self-renewing T cells (ISTCs), exhibit progressive phenotypic changes over time and stimulation history, but maintain their ability to proliferate and form durable memory. This finding challenges the previously held belief that T cell exhaustion, senescence or death are inevitable outcomes of the ageing process itself. Understanding the underlying biological mechanisms and metabolic pathways that allow for the everlasting proliferative capacity and longevity of T cells, such as ISTCs, may lead to new strategies for improving immune function and overall health in older adults.

## Conclusion

Recent studies have shown that metabolic dysregulation is a shared factor that links suboptimal immune responses in vulnerable cohorts, resulting in inferior vaccine outcomes. In our own opinion, the significance of nutritional intake and systemic metabolism in shaping vaccine responses might have been underestimated. Understanding the metabolic factors that contribute to optimal immune responses during vaccination is a growing area of research and has accelerated the field of immunometabolism. However, the extensive diversity in metabolites and immune cell types has made it difficult to identify causal relationships. Immunometabolism is at the crossroads of physiological status, nutritional regulation and immune outcomes. Therefore, a systems biology approach of multimodal profiling and mathematical modelling will be needed to unravel the fundamentals in such complex interactions.
